# A preliminary fMRI study of a novel self-paced written fluency task: observation of left-hemispheric activation, and increased frontal activation in late vs. early task phases

**DOI:** 10.3389/fnhum.2015.00113

**Published:** 2015-03-10

**Authors:** Laleh Golestanirad, Sunit Das, Tom A. Schweizer, Simon J. Graham

**Affiliations:** ^1^Harvard Medical School, Massachusetts General HospitalBoston, MA, USA; ^2^Keenan Research Institute, St. Michael's HospitalToronto, ON, Canada; ^3^Sunnybrook Research Institute, Sunnybrook Health Sciences CentreToronto, ON, Canada

**Keywords:** fMRI, language, verbal fluency, phonemic fluency, tablet, writing

## Abstract

Neuropsychological tests of verbal fluency are very widely used to characterize impaired cognitive function. For clinical neuroscience studies and potential medical applications, measuring the brain activity that underlies such tests with functional magnetic resonance imaging (fMRI) is of significant interest—but a challenging proposition because overt speech can cause signal artifacts, which tend to worsen as the duration of speech tasks becomes longer. In a novel approach, we present the group brain activity of 12 subjects who performed a self-paced written version of phonemic fluency using fMRI-compatible tablet technology that recorded responses and provided task-related feedback on a projection screen display, over long-duration task blocks (60 s). As predicted, we observed robust activation in the left anterior inferior and medial frontal gyri, consistent with previously reported results of verbal fluency tasks which established the role of these areas in strategic word retrieval. In addition, the number of words produced in the late phase (last 30 s) of written phonemic fluency was significantly less (*p* < 0.05) than the number produced in the early phase (first 30 s). Activation during the late phase vs. the early phase was also assessed from the first 20 s and last 20 s of task performance, which eliminated the possibility that the sluggish hemodynamic response from the early phase would affect the activation estimates of the late phase. The last 20 s produced greater activation maps covering extended areas in bilateral precuneus, cuneus, middle temporal gyrus, insula, middle frontal gyrus and cingulate gyrus. Among these areas, greater activation was observed in the bilateral middle frontal gyrus (Brodmann area BA 9) and cingulate gyrus (BA 24, 32) likely as part of the initiation, maintenance, and shifting of attentional resources. Consistent with previous pertinent fMRI literature involving overt and covert verbal responses, these findings highlight the promise and practicality of fMRI of written phonemic fluency.

## Introduction

Tests of spontaneous word generation, in which subjects are instructed to produce as many exemplars from a specified category as possible, are referred to as “verbal fluency” tasks and are among the most frequently used neuropsychological assessments to characterize various brain pathologies (Wolfe et al., 1987; Ruff et al., 1997; Stuss et al., 1998; Troyer et al., 1998; Henry and Crawford, 2004; Phillips et al., 2004). There are two major variants: phonemic fluency (e.g., “tell me all the words you can think of that begin with the letter A”) and semantic fluency (e.g., “tell me all the animals you can think of”). Performance on these tasks depends on the ability to organize words into meaningful “clusters,” and the flexibility to search and retrieve new clusters.

The neural correlates of verbal fluency are of substantial interest and have been extensively studied in the past few years (Indefrey and Levelt, 2000; Robinson et al., 2012; Wagner et al., 2014). Continuing to advance such investigations using functional magnetic resonance imaging (fMRI) is important, to refine the understanding of neuropsychological tests, and to execute clinical neuroscience studies that may eventually lead to medical applications involving the imaging modality. There are challenges to performing fMRI of verbal fluency, however. Traditional assessment of verbal fluency is undertaken by free recall of words, not from a learned list, but from long term memory (Birn et al., 2010) with subjects using overt speech to produce words as quickly as possible, over a typical timescale of 60 s. Unfortunately, overt speech generates task-correlated head and articulatory organ movements that have been shown in multiple studies to cause signal artifacts in the frontal lobe, impairing the ability to map language production areas (Birn et al., 1998, 1999; Huang et al., 2002; Gracco et al., 2005). Furthermore, typical fast image acquisition sequences used in fMRI generate loud acoustic noise (approximately 100–110 dB) that can obscure voice perception even when noise suppressing headphones are worn, and also can make it difficult to record overt responses quantitatively.

Previous fMRI studies have used modified verbal fluency tasks in attempts to circumvent these problems. Covert speech production has been the simplest, most common strategy employed, although covert speech cannot be recorded, verified and subjected to detailed behavioral analysis (Curtis et al., 1998; Schlösser et al., 1998; Lurito et al., 2000; Gurd et al., 2002; Gaillard et al., 2003; Weiss et al., 2003). Others have used more sophisticated fMRI acquisition techniques such as “clustered” sequences, in which a silent period is interleaved with the acquisition of brain images (Fu et al., 2002). This approach effectively suppresses the confounding effects of scanner noise, as well as the tissue motion and dynamic magnetic field distortion artifacts that arise from overt speech—but then the requirement of free recall of words from long term memory becomes compromised as subjects are required to produce words only during the pre-allocated silent periods. Very recently, researchers have utilized new protocols involving orthogonal microphones that enable scanner noise to be suppressed in relation to overt responses, and special fMRI data acquisitions that perform real-time adjustments to provide improved compensation for head motions during speaking as well as dynamic changes in magnetic field inhomogeneity (Katzev et al., 2013). However, these techniques are not yet common-place. Considering other alternatives, one interesting methodological option is to investigate the potential for studying fluency using another natural, extensively trained form of human communication—written responses.

Recently, our laboratory developed a novel computerized tablet and stylus that enables writing and drawing behavior to be studied during fMRI (Tam et al., 2011). Tablet fMRI experiments to investigate aspects of human motor control have demonstrated high quality activation maps in young healthy adults, without problematic task-correlated head motion (Callaert et al., 2011; Garbarini et al., 2013). As successfully shown in handwriting language production studies using electroencephalography (Perret and Laganaro, 2012), the use of a digitizing tablet permits the study of various language tasks through quantitative written responses. Thus, the fMRI-compatible tablet potentially provides an alternative, useful means of studying fluency without the challenges associated with fMRI of overt speech. The purpose of the present work, therefore, was to provide an example demonstration of this capability by performing a novel, preliminary proof-of-principle investigation in young healthy adults of the neural correlates of written phonemic fluency over a 1-min self-paced word generation by free recall from long term memory, analogous to standard behavioral test demands.

To our knowledge (and at least partly due to the reasons outlined above), no fMRI study has been performed yet that constitutes a direct attempt to measure the brain activity associated with long-duration self-paced versions of either oral or written phonemic fluency tasks. [However, the event-related potentials (ERPs) associated with speaking and writing have been studied recently during object naming, showing highly similar electrophysiological time-courses associated with conceptual and lexical-semantic processing (Perret and Laganaro, 2012)]. We hypothesized, therefore, that brain activity supporting written phonemic fluency test performance includes a distributed network highly similar to that reported in fMRI fluency studies involving overt and covert responses, involving the left anterior inferior frontal gyrus (L AIFG), the left middle frontal gyrus (L MidFG), the medial frontal gyrus (L MedFG) [Brodmann Areas (BA) 45, 46, and 9, respectively] (Phelps et al., 1997; Curtis et al., 1998; Dye et al., 1999; Hutchinson et al., 1999; Lurito et al., 2000; Fu et al., 2002; Abrahams et al., 2003; Halari et al., 2006), the precentral gyrus (BA 6) (Fu et al., 2002; Abrahams et al., 2003; Halari et al., 2006; Kircher et al., 2011), and the anterior cingulate (BA 24, 32) (Phelps et al., 1997; Dye et al., 1999; Fu et al., 2002; Halari et al., 2006). Brain regions in the left anterior inferior frontal gyrus (L AIFG) and the left medial frontal gyrus (L MedFG) are involved in strategic word retrieval (Yetkin et al., 1995; Costafreda et al., 2006; Snyder et al., 2007) whereas the activation of anterior cingulate reflects the attentional demands of verbal fluency tasks (Costafreda et al., 2006; Basho et al., 2007; Wagner et al., 2014). Left precentral gyrus, on the other hand, has been shown to have a role in preparing the coordination of complex articulatory movements prior to end-stage execution of speech commands (Baldo et al., 2011). Lesion studies also have revealed cases where discrete lesions confined to left precentral gyrus caused lexical agraphia (while the phonological system was relatively spared) (Rapcsak et al., 1988). Consequently, we predicted that the precentral gyrus would be activated during the written phoneme fluency task, playing a mediating role between strategic semantic, phonological/orthographical, and motor execution systems. As to the writing component of the task, it has been shown that several foci in posterior parietal cortex (PPC) and specifically the superior parietal lobule (SPL) are consistently activated during fMRI while writing with paper and a pencil (Segal and Petrides, 2012). In this preliminary study however, we wished to demonstrate that writing-specific activations can be suppressed in fMRI maps through use of an appropriate control task that mimics the act of hand-writing. Thus, no specific hypotheses regarding writing-specific activation loci were made.

An important behavioral observation in fluency tests is that words are typically generated most rapidly during the early recall phase (approximately the first 15 s). This period, when search and retrieval strategies are the most flexible, is typically thought to involve the frontal cortex and its role in executive functioning (Troyer et al., 1998; Schweizer et al., 2010; Arasanz et al., 2012). As time progresses after this phase, however (15–60 s), the rate of word production decreases as strategic flexibility weakens (Troyer et al., 1998). Thus, comparing test performance in the early phase vs. the late phase is often revealing. For example, verbal fluency has been extensively used in detection of Alzheimer's Disease (AD, Monsch et al., 1994; Mathuranath et al., 2000), with early phase performance similar to controls and AD-related impairments appearing in late phase performance (Birn et al., 2010). Thus, for the long-duration self-paced written paradigm developed in the present study, it was hypothesized that behavioral performance follows the same pattern as overt responses (i.e., fewer words are generated in late phase vs. early phase). In addition, it was hypothesized that declined output in the late phase of fluency is accompanied with increased brain activity in frontal regions that play a role in task initiation and maintenance, and shifting of attention resources.

## Materials and methods

### Subjects

Twelve young healthy adults with no history of neurological disorders participated in the study (6 male and 6 female; mean age 27 years; range 12 years). All subjects had normal or corrected-to-normal visual acuity. Ten subjects were native English speakers and the other two had extensively studied in English for more than 10 years and were fluent in both written and spoken English. The inclusion of non-native English speakers was based on previous studies of fluency task reporting no significant difference in the number of words produced in 1 min between native English speakers and fluent non-native speakers (Grogan et al., 2009). Moreover, inclusion of bilingual speakers has clinical relevance as they are more representative of the human population, which typically speaks more than one language (Wei, 2000).

Handedness was evaluated by the Edinburgh Handedness Inventory (Oldfield, 1971). Eight subjects were evaluated as right-handed (mean ± standard deviation handedness score 78.75 ± 24.57), three subjects were evaluated as left-handed (mean ± standard deviation handedness score −81.33 ± 26.39) and one subject was ambidextrous (handedness score −26).

Although many fMRI studies report group brain activity from a population of right-handed individuals, assuming that left-handed individuals have different spatial organization of brain function than their more common, right-handed counterparts, the dependence of language laterality on handedness is not absolute. Previous work has shown definitively that the majority of strongly left-handed subjects still exhibit left-lateralized language processing (Knecht et al., 2000). Thus, for expediency and to increase the statistical power to detect brain activity in this preliminary work, it was decided that including a small number of left-handed or ambidextrous individuals was acceptable if and only if they displayed left-lateralized language processing based on a test of fMRI language laterality conducted prior to written phonemic fluency (see below). On this basis, all subjects mentioned above were fully included in the written phonemic fluency data collection. Furthermore, we included a control task that was intended to subtract out the activation associated with hand-writing movement (see below). All subjects provided their free and informed consent to participate in the study, which was approved by the Research Ethics Board at Sunnybrook Health Sciences Centre.

#### Tablet technology and stimuli projection setup

**Figure 1:**
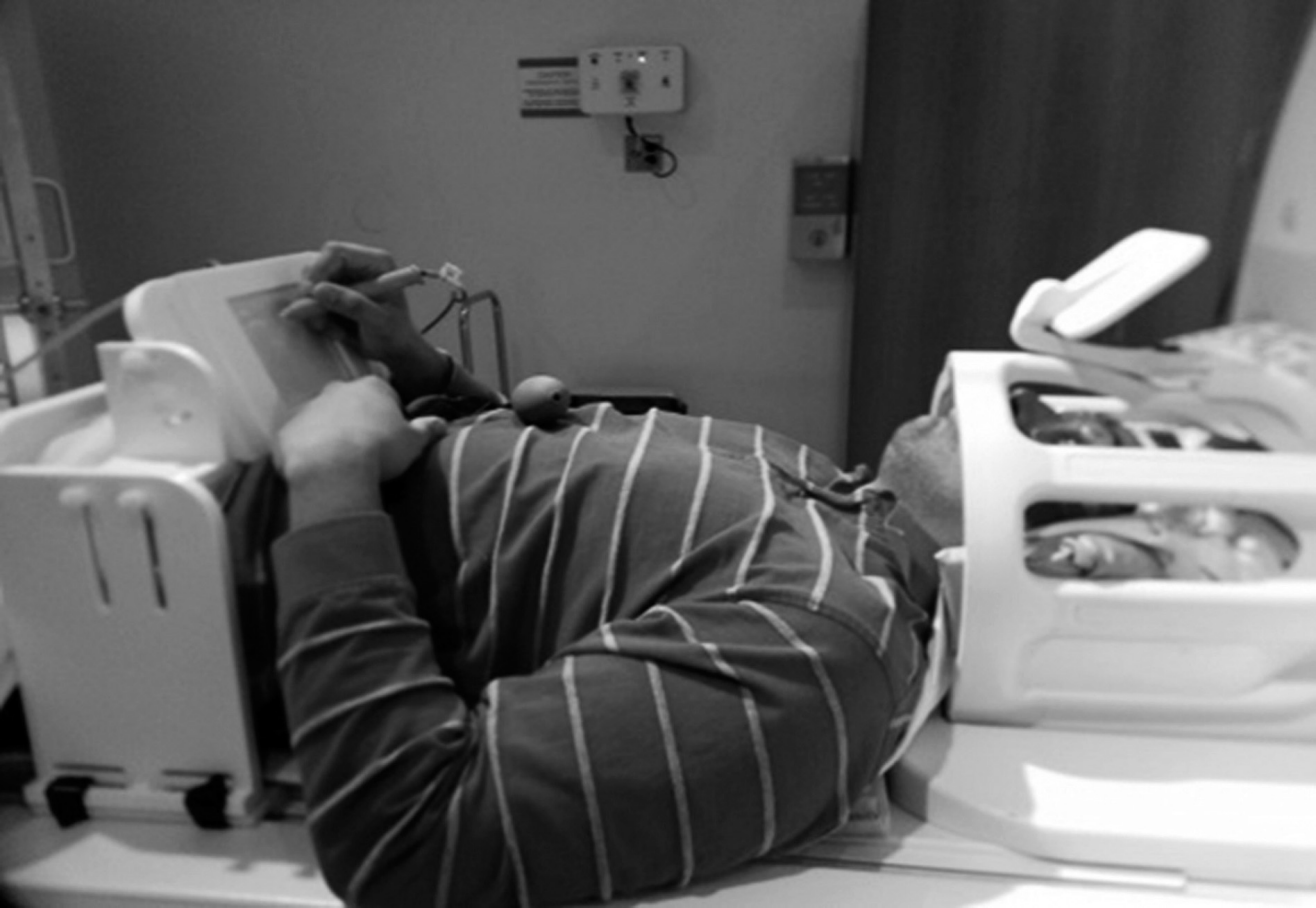
**The fMRI-compatible tablet mounted for use by a subject**. An angled mirror mounted on the head coil was used to view visual stimuli on a rear projection screen (not shown).

The tablet system (see Figure 1) included a touch-sensitive screen, a support platform, a stylus and a controller box, as well as the necessary software and cabling to record responses and provide task-related feedback. Detailed hardware validation has been reported previously (Tam et al., 2011). The support platform was constructed of plastic and featured a tilting stage of adjustable height to accommodate users comfortably in the limited space available in the magnet bore, while keeping the writing surface off the torso and reducing interference from respiratory motion. The tablet and stylus signals passed through an electromagnetic interference filter (56-705-005-LI, Spectrum Control, Fairview, PA) at the penetration panel and through shielded cables to the tablet controller box in the operator console area. The controller box contained the touch screen controller board, power conditioner, and receptacles for universal serial bus (USB) connections to the fMRI stimulus/response computer. Software on the computer interpreted the tablet and/or stylus input to provide task-related feedback while also recording detailed logs of behavior for subsequent analysis. For this study, touching the stylus to the tablet would result in “ink” marks at the analogous locations on the display, resembling a pen-and-paper task. Stimulus presentation was programmed and controlled with E-Prime Software 2.0 (Psychology Software Tools, Sharpsburg, PA; task programs available upon request to S.J.G.). Visual stimuli were presented to the subject using an MRI-compatible projector (Silent Vision, Avotec Inc., Stuart, FL) and backprojection screen located at the rear of the magnet bore (20° × 15° visual angle), viewed through an angled mirror mounted on the head coil. Written responses were recorded as tablet x,y coordinates as a function of time, in data files for further processing.

#### Experimental tasks

At the outset, careful methodology was applied to ensure that the tablet could be used by subjects comfortably and proficiently. On the day of the experiment, all subjects first completed 5–10 min of familiarization with tablet and stylus outside of the scanner to copy sample words. Subjects sat on a chair with the tablet on their lap. A series of words were represented on a monitor and subjects were instructed to use the tablet and stylus to copy the words. Performance was self-paced, with subjects required to click on the “Next” button at the bottom of the page after copying a word (similar to the written phonemic fluency task, see below) to clear the screen and advance to the next word copying trial. The familiarization period helped to assure that all subjects became completely comfortable with the tablet and used it with the same ease as when using a pen and paper. The familiarization period was administered for each subject until they reached a reasonable pace of approximately 4 s per word.

After familiarization with the tablet, subjects had a brief rest (approximately 10 min) and then were asked to practice once more by performing a 1-min written phonemic fluency task with the same timing and priming as the actual test (see below). The practice was conducted using the letter “N,” a letter that was not included during fMRI. This practice session confirmed that all subjects fully understood the task instructions and could perform the written phonemic fluency test successfully.

Inside the scanner, great care was taken to ensure that tablet height and orientation were adjusted within the magnet bore so that subjects were able to perform ergonomic stylus/tablet interactions. After tablet adjustment, subjects first repeated the word copy task used in the familiarization period with 20 words, under instructions to use the tablet while keeping their head and shoulders as still as possible. As judged by the task administrator (L.G.), all subjects performed with the same level of performance (approximately 4 s/word) as they did outside of scanner, indicating that writing performance inside the scanner was not significantly influenced by the supine position of the subjects.

**Figure 2:**
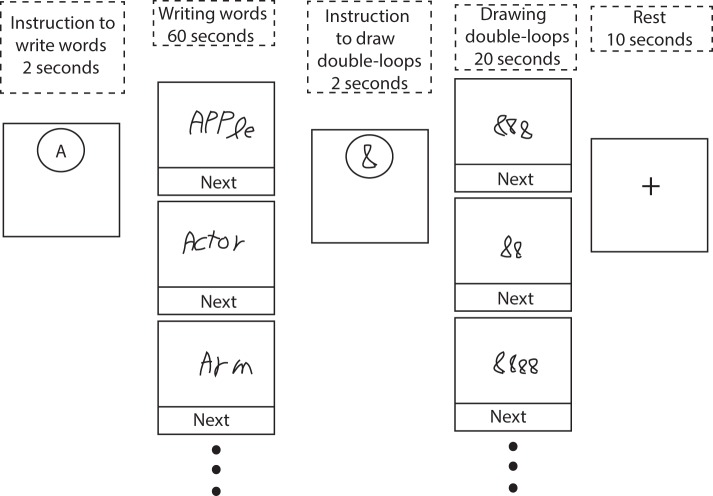
**Schematic of the phonemic fluency task involving written responses, and a control condition consisting of drawing symbol strings composed of double-loops**. At the beginning of each block, subjects were presented with a 2 s instruction image. Subjects received real-time visual feedback of their hand-writing during task performance. See text for further details.

The written phonemic fluency task (Figure 2) was administered as a block design consisting of repetitions of a 60 s task block, a 20 s control block, and a 10 s rest interval. In addition, a 2 s instruction slide was presented prior to task and control blocks. Subjects were presented with a cue letter (either F, A, S, D, or C) that was projected on the screen for 2 s with instructions to write down as many words as possible that started with the cue. Letters F, A and S are the most commonly used cues in clinical phonemic fluency tests (Strauss et al., 2006), based on the frequency of occurrence of English words. The two other letters (C and D) were chosen as the English word frequency is similar to that of the previous set (Mayzner and Tresselt, 1965). Subjects were instructed not to repeat words within a given task block, not to use suffixes as word generation strategy, and not to write proper names. A “Next” box was presented on the bottom of the screen that subjects pressed after writing each word to refresh the screen before supplying the next word. This procedure also served to eliminate any effects on word generation introduced by viewing previous words on the display.

To control for neural activities associated with early motor and visual components of the task, general executive activity due to arousal and attention, and for regions of brain activity specifically engaged by the action of writing, subjects were required to perform a 20 s control task during which they drew symbol strings composed of double-loops. That is, they were instructed to draw “8,” “88,” “888,” etc., based on their own choice. This task was designed to mimic the motor and visual activity of normal handwriting and screen refreshing without engaging any substantial linguistic or memory components (Segal and Petrides, 2012). The control block was followed by a 10 s rest period with a fixation cross presented in the middle of the screen.

The fMRI rhyming task used for evaluating language laterality was similar to that described in Salvan et al. (2004). The task presented visually rhyming and non-rhyming word pairs. The control condition presented paired bar patterns that matched or did not match. Eight task and control blocks were presented with six stimuli in each block (with a stimulus duration of 3 s). In the task condition, subjects were required to make a forced-choice decision whether words rhymed or did not rhyme by a touch response on the tablet, corresponding to one of two locations on the display screen. The control condition required the same response mechanism to determine whether the bar patterns matched or did not match. Word pairs contained a mix of words that were spelled similarly and rhymed (e.g., bike, hike); were spelled similarly and did not rhyme (e.g., blood, hood); were spelled differently and rhymed (e.g., here, fear); and were spelled differently and did not rhyme (e.g., breed, bread). This methodology ensured that the subject had to perform careful silent reading to perform well on the task. The pattern of activation seen during rhyming (data not shown for brevity) has been reported by others to be more specific for Wernicke's and Broca's area in comparison to typical fluency or word generation tasks, and thus is suitable for laterality analysis.

Language laterality was assessed by calculating a commonly used laterality index (LI) (Binder et al., 1996; Seghier, 2008):

(1) $$LI=\frac{Q_{LH}-Q_{RH}}{Q_{LH}+Q_{RH}},$$

where *Q_LH_* and *Q_RH_* represent the number of active voxels for the left hemisphere and right hemisphere contributions, respectively, focusing specifically on regions of interest (ROIs) within the posterior inferior frontal gyrus (Broca's area, BA 44) and the posterior superior temporal gyrus (Wernicke's area, posterior part of BA 22). Previously, LI values obtained with these ROIs were found to correspond better with Wada language laterality test results than LI values obtained from whole hemisphere calculations (Spreer et al., 2002). Anatomical landmarks corresponding to left Broca and Wernicke areas and their right hemisphere homologous (right Broca and right Wernicke, hereafter) were manually defined on anatomical images transformed into Talairach coordinates by an experienced neurologist. ROIs were then drawn on a locked view of functional data for voxel counting. We calculated *Q_LH_* as the sum of activated voxels in the left Broca and left Wernicke regions (*L_Broca_* + *L_Wernicke_*), and similarly, *Q_RH_* as the sum of activated voxels in the right Broca and right Wernicke areas (*R_Broca_* + *R_Wernicke_*). Accordingly, our laterality index was calculated as:

(2) $$LI=\frac{\left(L_{Broca}+L_{Wernicke}\right)-(R_{Broca}+R_{Wernicke})}{\left(L_{Broca}+L_{Wernicke}\right)+(R_{Broca}+R_{Wernicke})}$$

Subjects with a conservative threshold of LI > 0.25 (Baciu et al., 2005) were evaluated as left-dominant.

#### MRI acquisition and data analysis

Functional MRI was conducted at 3.0 T using a research-dedicated system (MR750, GE Healthcare, Waukesha, WI) using a standard 8-channel head coil receiver. Foam padding was placed under arms and elbows to add comfort if requested, as part of ensuring that subjects performed with the tablet to the best of their abilities. Head movement was minimized through use of foam cushions and a band of surgical tape affixed to the forehead and head coil to enhance the sensation of head motion for the subject by tactile feedback. High-resolution anatomical imaging (axial 3D FSPGR, *TI* = 650 ms, field of view (FoV) = 22 cm × 16.5 cm, flip angle (FA) = 8°, matrix = 256 × 192, 1.0 mm thickness, 190 slices) was acquired prior to blood oxygenation level-dependent (BOLD) fMRI. Functional MRI was undertaken using axial 2D T2^*^-weighted spiral in-out k-space trajectories (*TE* = 30 ms, *TR* = 2000 ms, *FA* = 70° FoV = 20 cm × 20 cm, 64 × 64 matrix, 4.5 mm thickness, 30 slices) (Chang and Glover, 2011).

Data for each subject were acquired in two fMRI runs, separated in time by about 10 min due to fMRI of other behavioral tasks as part of a larger test battery (data not reported here). Each run contained three block procedures over a time of approximately 5 min. In the first run, subjects wrote words starting with each of the letters F, A, or S. In the second, subjects wrote words starting with letters D, C, and S. Although there was a potential for learning effects (and associated spatiotemporal modulations in brain activity) associated with performing a second instance of written phonemic fluency with the letter S, the decision to include this additional task block was made with the desire to increase statistical power as part of a proof-of-principle, preliminary report. The impact on brain activity by repeating the S task was judged to be minor for several reasons: (a) the frequency of words that start with the letter S is close to the mean frequency of the other letters; (b) the range of frequencies associated with words starting with A, F, D, and C already was expected to vary task demands slightly over each task block; and (c) the learning effects associated with the second repetition of the S task were expected to be minor in relation to subject-to-subject variations in task performance, over the relatively small but reasonable cohort size studied in this preliminary work. Furthermore, the potential for learning effects on the second S task block was mitigated partly by experimental design and partly by how subjects were instructed. The second S task was placed at the end of the fMRI session, with other cognitive tasks (part of a larger test battery) providing interference over a timeframe of approximately 10 min. In addition, subjects were told to treat the second instance of the S task as a “new run.” That is, they were instructed that they did not need to remember, or avoid words starting with the letter S that they wrote during the first instance.

Functional MRI data were analyzed using Analysis of Functional NeuroImages (AFNI) software (Cox, 1996). The first five volumes of each functional run were discarded to eliminate the fMRI signal decay associated with magnetization reaching equilibrium. The remaining fMRI data were temporally interpolated for slice time correction, co-registered to the third time point of the first run for motion correction, and spatially smoothed with a 6-mm full width-at-half-maximum (FWHM) Gaussian kernel. Two statistical parameter maps were generated using a General Linear Model (GLM). First, activation maps contrasting written phonemic fluency (PF) for the entire 60 s block duration vs. 20 s control condition of drawing double loops (DDL) were produced and investigated to verify if general neural correlates reported in previous covert and overt studies of fluency tasks were also present in the long-duration written version of the task. Second, activation maps contrasting the first 20 s (PF_first20) vs. the last 20 s of the task (PF_last20) were produced to investigate whether there was a substantial difference between neural correlates active during the early phase and late phase of written phonemic fluency. Instead of characterizing brain activity for the early and late phases by assessing the first 30 s and last 30 s of the task, as reported for behavioral performance (see below), shorter 20 s durations were compared to eliminate the possibility that the sluggish BOLD hemodynamic response from the early phase would affect activation estimates from the late phase. For both maps, GLM analyses included boxcar waveforms convolved with a gamma function representative of the BOLD hemodynamic response function, and run-wise third order Legendre polynomials and six-degree-of-freedom head motion estimate parameters as nuisance covariates. The GLM was solved using least squares fitting of the data to produce estimates of effects (beta coefficients) and their standard errors, as well as t-statistics for each comparison of interest.

Subsequent to the first-level analysis of individual subjects described above, anatomical images were aligned to the third time point of the first fMRI run and then transformed to Talairach space (Talairach and Tournoux, 1988) based on the AFNI TT_N27 brain template using piece-wise affine transformation. The same transformation was applied to the individual activation maps including linear interpolation to a 2 × 2 × 2 mm voxel grid. The beta coefficient map from each subject was spatially smoothed with an 8 mm FWHM Gaussian kernel to compensate for inter-subject variance in anatomical structure. Group activation maps were created with a random-effects model, treating subjects as the random factor. A single-sample, two-tailed *t*-test was then conducted at each voxel for each run to identify voxels with mean beta coefficients that differed from zero. The group maps were thresholded using a voxel-wise 2-tailed probability with false discovery rate (FDR) correction for multiple comparisons at corrected *p* < 0.05.

## Results

### Behavior

Overall, subjects performed the tasks consistently well and without obvious difficulty. Motion parameters estimated from volume registration were visually inspected to ensure that head motion was not confounding fMRI results. All subjects completed the task with negligible peak head motion (<0.5 mm displacement along any Cartesian axis direction).

**Figure 3:**
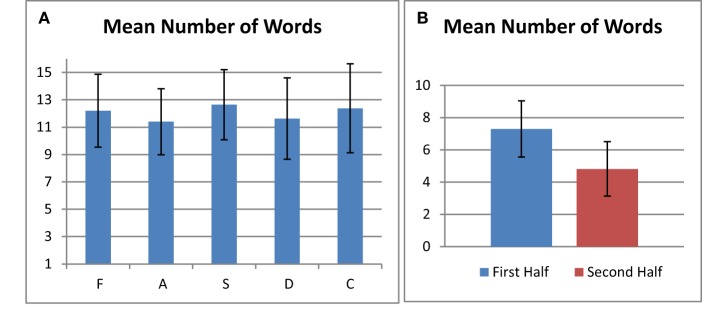
**(A)** Number of generated words for each letter averaged over all subjects. **(B)** Number of generated words in the first half and second half of the test averaged over all letters and all subjects. In both plots, error bars represent standard deviation of the mean.

Subjects generated 12.1 ± 2.7 words per minute (mean ± standard deviation, calculated over all subjects and all letters), excluding repeated words and incomplete trials, and 6.0 ± 2.1 (mean ± standard deviation) double-loop strings per 20 s. Figure 3A shows the mean number of words written for each letter separately. It is evident that performance across the cohort was very similar over all letters with respect to both mean and standard deviation. In particular, the pooled results for written phonemic fluency involving the letter S, which subjects performed twice, were not distinctive in relation to performance involving the other letters. Figure 3B shows that subjects produced significantly more words in the first half (30 s) of the phonemic fluency test compared to the second half of the test (paired two-tailed *t*-test, first half mean number of words ± standard deviation 7.3 ± 1.7, second half mean number of words ± standard deviation 4.8 ± 1.7, *p* < 0.0001). One subject produced a substantially smaller number of words per letter (mean number of words ± standard deviation 4.6 ± 1.0) and was excluded from brain mapping analysis of PF_first20 vs. PF_last20 as a consequence.

### Laterality indices and handedness scores of left-handed and ambidextrous subjects

**Figure 4:**
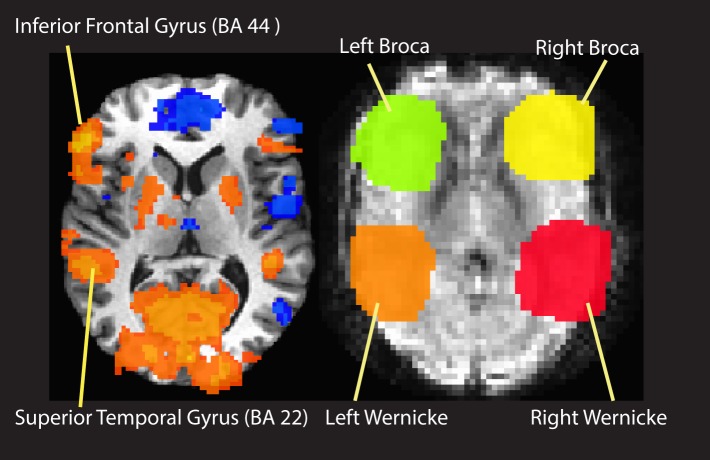
**An example of ROI definition for calculation of laterality index**.

Figure 4 gives an example showing how ROIs were defined on activation maps of the Rhyming task. Table 1 reports active voxel counts in left and right Broca and Wernicke regions for four subjects whose handedness scores were below 40. The resulting LI's were all greater than the conservative cutoff value that was pre-set at 0.25. Consequently, these four left-handed and ambidextrous subjects were included in the main analysis.

**Table 1:** **Details of handedness scores and laterality indices for left-handed and ambidextrous subjects**.

**Voxel count**					
**LBroca**	**R Broca**	**L Wernicke**	**R Wernicke**	**LI**	**Handedness score**
21	2	59	0	0.95	−100
136	47	104	33	0.50	−44
145	65	66	51	0.29	−100
89	37	76	58	0.26	−26

### Written phonemic fluency vs. drawing double loops

**Figure 5:**
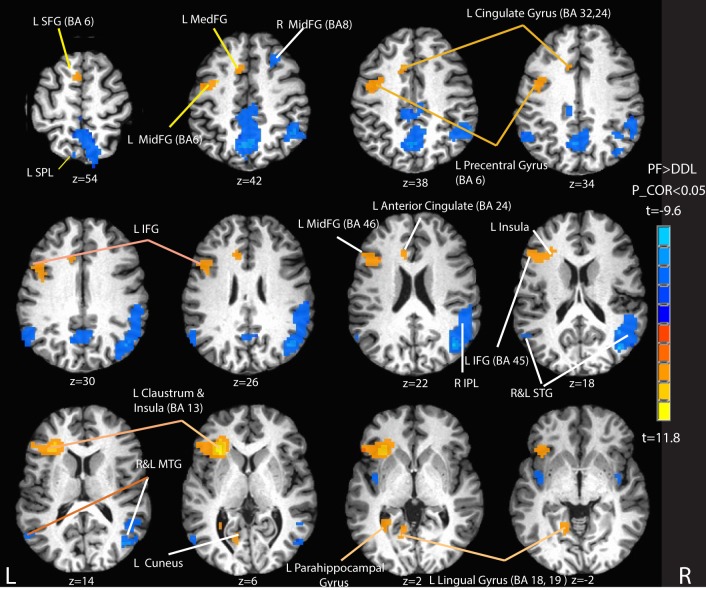
**Selected activation maps for written phonemic fluency (PF) vs. the control condition of drawing double loops (DDL)**. Orange and yellow areas represent Phonemic Fluency > Control activation. Blue areas represent Control > Phonemic Fluency activation. Peak activation foci are reported in Table 2. L SFG, left superior frontal gyrus; L SPL, left superior parietal lobule; L MedFG; left medial frontal gyrus; L MidFG, left middle frontal gyrus; R MidFG, right middle frontal gyrus; L IFG, left inferior frontal gyrus; R IPL, right inferior parietal lobule; R and L STG, right and left superior temporal gyrus; R and L MTG, right and left middle temporal gyrus. P_COR, false discovery rate corrected *p*-value.

Figure 5 and Table 2 summarize the brain activity for 60 s of written phonemic fluency (PF) contrasted with the 20 s control task of drawing double loops (DDL). Robust positive contrast attributable to the PF condition (Written Phonemic Fluency > Control, shaded in orange and yellow in Figure 5) was largely confined to the left hemisphere, in regions such as the left superior frontal gyrus (BA 6), left middle frontal gyrus, left medial frontal gyrus, left precentral gyrus, left anterior inferior frontal gyrus, left claustrum and insula, and the anterior cingulate. Positive activation was also observed in the left cuneus, left lingual gyrus, and left parahippocampal gyrus. Negative contrast attributable to the DDL task (Control > Written Phonemic Fluency, shaded in blue in Figure 5) yielded greater bilateral and right hemisphere activity, including the bilateral superior and middle temporal gyri, the right inferior parietal lobule and the right middle frontal gyrus. Negative contrast attributable to the DDL task was also observed in the left superior parietal lobule.

**Table 2:** **Peak of activations for written phonemic fluency vs. control (draw double loops)**.

**Location**	**Cluster size (voxels)**	**t-statistics**	**MNI coordinates (mm)**		
**(A) PHONEMIC FLUENCY > CONTROL**					
**LEFT HEMISPHERE**					
Insula	254	11.7	−27	23	10
Anterior cingulate	47	7.0	−8	23	24
Cuneus	23	5.2	−11	−67	5
Parahippocampal gyrus	11	5.8	−27	−51	1
**(B) CONTROL > PHONEMIC FLUENCY**					
**LEFT HEMISPHERE**					
Precuneus (BA 7)	407	−7.7	−2	−63	39
Supramarginal gyrus	48	−6.4	−56	−59	30
Insula	15	−5.9	−39	−2	−1
Superior parietal lobule	10	−6.9	−17	−70	68
Middle temporal gyrus	10	−5.9	−56	−61	10
**RIGHT HEMISPHERE**					
Middle temporal gyrus	396	−9.7	39	−68	19
Superior temporal gyrus	27	−6.7	53	2	−12
Middle frontal gyrus	11	−6.0	27	21	49

*MNI, Montreal Neurological Institute*.

### Written phonemic fluency: last 20 s vs. first 20 s

**Figure 6:**
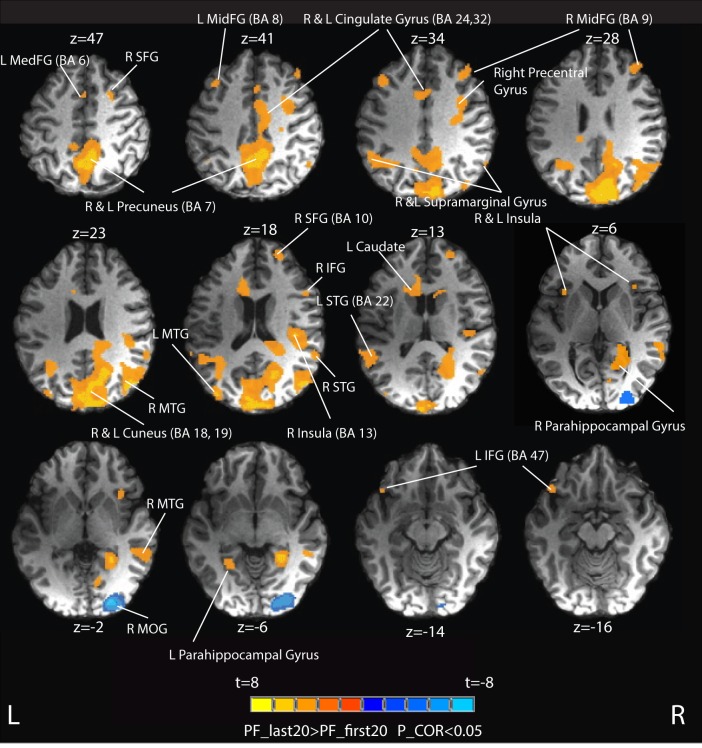
**Group activation maps contrasting the last 20 s of written phonemic fluency (PF_last20) vs. the first 20 s (PF_first20)**. Orange areas (the majority) represent PF_last20 > PF_first20. Blue areas represent PF_First20 > PF_last20. Peaks of activation foci are reported in Table 3. L MedFG, left medial frontal gyrus; R SFG, right superior frontal gyrus; L MidFG, left middle frontal gyrus; R MidFG, right middle frontal gyrus; R MTG, right middle temporal gyrus; L MTG, left middle temporal gyrus; R STG, right superior temporal gyrus; R IFG, right inferior frontal gyrus; L STG, left superior temporal gyrus; R MTG, right middle temporal gyrus; L IFG, left inferior frontal gyrus; R MOG, right middle occipital gyrus.

Figure 6 and Table 3 summarize the brain activity for contrasting the last 20 s of written phonemic fluency (PF_last20) vs. the first 20 s (PF_first20).

**Table 3:** **Peak of activation contrasts for early-phase (PF_first20) and late-phase (PF_last20) written phonemic fluency**.

**Location**	**Cluster size (voxels)**	**t-statistics**	**MNI coordinates (mm)**		
**PF_last20 > PF_first20**					
**LEFT HEMISPHERE**					
Cuneus (BA 18)	5997	8.0	−7	−95	12
Superior temporal gyrus (BA 22)	705	4.6	−54	−45	13
Caudate	299	4.9	−11	19	18
Middle frontal gyrus	109	5.0	−41	22	43
Middle temporal gyrus	97	5.1	−35	−82	17
Parahippocampal gyrus (BA 19)	58	4.7	−25	−48	−5
Inferior frontal gyrus	31	5.7	−41	29	−15
Cingulate gyrus	21	3.9	−17	−31	29
Insula	15	3.9	−33	15	5
Medial frontal gyrus	13	3.9	−17	2	59
**PF_first20 > PF_last20**					
**RIGHT HEMISPHERE**					
Middle temporal gyrus	650	6.6	41	−74	22
	166	4.2	54	−38	0
Insula	301	5.0	37	−25	20
Middle frontal gyrus (BA 8)	170	4.9	41	30	45
Superior frontal gyrus (BA 10)	76	4.4	21	56	23
Superior temporal gyrus	54	4.0	58	−45	19
Medial frontal gyrus (BA 10)	11	4.6	9	61	−4
Middle occipital gyrus	420	−7.3	23	−86	−3

*MNI, Montreal Neurological Institute*.

Extensive positive activation (PF_last20 > PF_first20) attributable to the last 20 s of the task was observed. Areas of these positive activations (shaded in orange in Figure 6) included bilateral activation in precuneus, cuneus, middle frontal gyrus (MidFG, BA 9), insula, cingulate gyrus (BA 24, 32), parahippocampal gyrus, superior temporal gyrus (STG) and middle temporal gyrus (MTG). Activation was also observed in right pre-central gyrus, right superior and inferior frontal gyri, left inferior frontal gyrus (BA 47), and left caudate. Greater activation was also observed in the bilateral supramarginal gyrus. Negative activation (PF_first20 > PF_last20, shaded in blue in Figure 6) attributable to the first 20 s of the task was observed only in right middle occipital gyrus.

## Discussion

This study provides a proof-of-concept example demonstration of how fMRI-compatible, computerized tablet technology can be usefully applied for mapping brain activity related to language production involving written responses. The example task that was developed and investigated was written phonemic fluency, designed in an analogous manner to clinical verbal fluency tests conducted in an office setting with overt speech (Ruff et al., 1997; Stuss et al., 1998; Troyer et al., 1998). The task was chosen due to its requirements for free-recall of words from long term memory over the relatively long time duration of 60 s—a design that would be challenging to undertake in an fMRI study with spoken responses due to motion-related signal artifacts associated with speech articulation (Birn et al., 1998, 1999; Huang et al., 2002; Gracco et al., 2005). Prior fMRI studies of phonemic fluency have either used tasks that were limited to short block durations (Phelps et al., 1997; Curtis et al., 1998; Dye et al., 1999; Lurito et al., 2000; Fu et al., 2002; Birn et al., 2010; Krug et al., 2011) or used covert word generation schemes lacking quantitative behavioral recording and analysis (Curtis et al., 1998; Schlösser et al., 1998; Lurito et al., 2000; Gurd et al., 2002; Gaillard et al., 2003; Weiss et al., 2003). In the present work, robust fMRI data were obtained while subjects interacted with the tablet without introducing problematic levels of head motion and magnetic field distortion. Written phonemic fluency performance, the underlying neural circuitry, and the larger ramifications of the work are discussed below.

### Written phonemic fluency performance

In the written version of phonemic fluency developed in the present study, subjects were required to write a word and then to perform a screen clearing operation before providing their next response. It might be anticipated that this procedure would slow the rate of word production in comparison to that observed with overt responses. In Figure 3, the average number of written words produced in each 60 s task period was approximately 12 for each of the 5 letters tested. Thus, the total number of “FAS” words over 3 letters was approximately 36. This number compares very favorably with the normative spoken phonemic fluency data for native English speakers of the same age which has been reported to be approximately 41 (Tombaugh et al., 1999; Troyer, 2000). Thus, over the 60 s task period, the slowing effect of providing written responses appears to be relatively small. This result also suggests a) that the cognitive loads required to keep words “on-line” in orthographical representations for hand-writing production and phonological representations for oral production while responding are not likely to be highly different; and b) that the 60 s task period is appropriate for written responses. The strength of these statements, which should be considered hypotheses, will need to be tested in future work that specifically includes investigation of patient populations, and that investigates how vocal and written phonemic fluency evolve over time within the 60 s task period.

### Phonemic fluency task vs. drawing double loops

As expected, written phonemic fluency yielded an activation map that was highly similar to previously reported covert and overt speech studies. Greater activation was observed in the left superior frontal gyrus (BA 6) as well as widespread activation starting from the left precentral gyrus extending along the left anterior inferior frontal gyrus to the left insula (see Figure 5), consistent with claims that these left hemisphere sites are involved in strategic lexical and semantic search and retrieval processes (Birn et al., 2010), and production. As mentioned earlier, activation in the left anterior IFG and left middle and medial frontal gyri (BA 45, 46, and 9) is likely associated with strategic semantic search processes whereas the activation of the anterior cingulate reflects the attentional demands of verbal fluency tasks.

Activation of the left parahippocampal gyrus was also observed in the present work, in agreement with previous reports observing parahippocampal activity in fluency tasks with high demand (e.g., comparing fluency for difficult letters vs. easy letters) (Fu et al., 2002; Halari et al., 2006).

At the outset, the fMRI experiment was designed with the expectation that, using a control task that strongly represents the act of writing (drawing symbol strings in the form of double loops), it would be possible to obtain activation maps of written phonemic fluency that were not substantially affected by the mode of response. Therefore, no specific hypotheses were given regarding activations specific to writing. In retrospect, however, the written phonemic fluency task and the DDL control task were found not to be balanced in terms of tablet performance demands. Subjects executed the DDL control task at a higher average rate (one symbol string every 3 s) than they performed written phonemic fluency (one word every 5 s). Consistent with this increased pace, a number of brain regions including left superior parietal lobule (SPL) and right middle and superior temporal gyri showed enhanced activity for the DDL control task relative to written phonemic fluency. The enhanced activation in left SPL is expected given its importance for written production (Alexander et al., 1992; Henderson, 1992). Specifically, enhanced activation was found in BA 7 that extended along the right intraparietal sulcus and into the right middle occipital gyrus. These areas support generation of the correct sequence of movements required for handwriting (Alexander et al., 1992; Sakurai et al., 2007), production of typing motor sequences (Gordon et al., 1998) and typed spelling (Purcell et al., 2011).

Returning to the written phonemic fluency task, interestingly, other activated regions shown in Figure 5, including a large region of posterior temporal– parietal cortex centered on the supramarginal gyrus, were lateralized to the right hemisphere. Birn et al. (2010) reported very similar activation in an overt fMRI study of fluency, contrasting “automatic speech” vs. fluency tasks. Repeated response of the same highly over-learned sequence of words (analogous to the control task in the present work) led to enhanced right hemisphere activity relative to considerably more effortful tasks requiring the generation of a unique list of words on every trial. One plausible interpretation is that these observations reflect a right hemisphere superiority for automatic speech production, consistent with some clinical and functional neuroimaging literature (Larsen et al., 1978; Code, 1997). However, other interpretations are possible and it should be recognized that the observed hemispheric differences could reflect any of the ways that the written phonemic fluency and the control conditions differed in this study.

### Late vs. early phases of written phonemic fluency

Much of neuropsychological value of fluency tests comes from their recruitment of multiple executive functions, namely, initiation, planning, purposeful action, self-monitoring and self-regulation, inhibition and flexibility (set-shifting). The long duration of the written phonemic fluency task studied in the present work allowed for direct comparison of neural components active during the early phase vs. those active during the late phase. As hypothesized, the late phase of the written fluency task was associated with greater brain activity in several predicted regions. Specifically, compared to the early phase (first 20 s), the late phase (last 20 s) produced robust activation in the bilateral middle frontal gyrus (BA 9) and the bilateral cingulate gyrus (BA 24 and BA 32) (see Figure 6). These areas are thought to support “energization” of a cognitive task, that is, the process of initiation and sustained purpose, and patients with lesions in these areas show disproportionate declines in word production during the last 45 s of the clinical letter fluency task compared with the first 15 s (Alexander et al., 2005, 2007; Shallice et al., 2007). Specifically, left anterior cingulate gyrus has been shown to play a strong role in maintaining goal-directed behaviors, particularly those that require the suppression of external or internal interfering influences (Pardo et al., 1990; Corbetta et al., 1991; Bench et al., 1993). The anatomical location of maximal cingulate activation in our study (BA 32, MNI coordinates −5, 13, 38) corresponds closely with that of a previous study involving encoding and retrieval of auditory–verbal memory (Fletcher et al., 1995). As performance of the written fluency task progresses over time, it may be that the increasingly onerous requirement not to repeat words particularly requires engagement of the anterior cingulate. Lesion studies also have shown the role of medial cortices in task-switching and error control (Shallice et al., 2007), processes that are more present in the late phase of written fluency, as words within one cluster are exhausted and there is a need to move to another cluster.

Interestingly, no distinct frontal areas showed greater activation in the early phase of written phonemic fluency compared to the late phase. Such activations might be expected, given previous work supporting the role of frontal cortex in supporting flexible search and retrieval strategies (Troyer et al., 1998; Schweizer et al., 2010; Arasanz et al., 2012; Ladowski et al., 2014). At present, the absence of these activations is difficult to explain, although it is possible that the approach of using written responses attenuates the efficiency of phonemic fluency in the early phase. Another possible explanation is that the GLM analyses employed in this preliminary fMRI study were insufficiently sensitive to provide a full characterization of spatiotemporal BOLD signals associated with early phase performance effects. A carefully undertaken fMRI study that compares written and overt phonemic fluency responses in the same subjects using multivariate analysis methods would be a solid approach to investigate and resolve this issue. At this stage, we also do not have an explanation for the activity observed in the right middle occipital gyrus in the early vs. late phase of phoneme task.

Nevertheless, as has been demonstrated with overt responses, phonemic fluency with written responses has the potential to provide clinically useful information about temporal differences in neural processing throughout the duration of the task. For example, schizophrenic patients produce significantly fewer words in the 3 min categorical verbal fluency task (Allen et al., 1993) and have significantly impaired “switching” and “clustering” strategies (Robert et al., 1998). Short duration forced-paced PET (Frith et al., 1995) and fMRI (Curtis et al., 1998) studies of fluency in schizophrenic patients have revealed differences in patterns of frontal and temporal activation compared to healthy controls. A self-paced long duration study of fluency, such as the one developed in this study, could provide critical information regarding differences in regional brain activity associated with different task strategies. Fluency tasks also differentiate AD patients from normal controls (Monsch et al., 1992; Henry et al., 2004) with behavioral differences mostly manifested during the late phase of the task. Investigating neural correlates of such differential responses in a long-duration written version of the fluency test could potentially provide useful etiological information.

### Limitations

It is important to place the results of this preliminary study in appropriate context by discussing a number of trade-offs and limitations in the chosen experimental design and approach. First and foremost, the strategy to assess phonemic fluency by written responses is expeditious from the standpoint of fMRI data acquisition and fMRI data quality, highlighting the utility of the computerized tablet to obtain useful activation maps related to language processing without substantial levels of motion artifact associated with overt responses. However, the ramifications of proceeding in this fashion should be considered carefully. In neuropsychological testing, verbal fluency testing with spoken responses is highly advantageous because of the ease of administration to a wide population. Although written responses are extensively practiced and learned by humans, overt speech is the more natural, intrinsic means of language communication. Individuals with awkward handwriting (perhaps due to lack of training or disuse) or with writing impairments, such as dysgraphia or writer's cramp, would have difficulty performing written phonemic fluency even if their language processing capabilities were fully intact. Furthermore, patient populations for which fMRI of phonemic fluency is of interest (e.g., stroke survivors) could also show deficits in fluency that interacts with writing production (or speech production). It is possible that for such individuals, the performance of phonemic fluency during fMRI with overt and written responses could help to characterize their brain and behavioral deficits more fully. Such a comparative study faces a number of methodological challenges, however, as indicated below.

Second, fMRI and behavioral results for free-recall written phonemic fluency are shown in this study that are very similar to literature reports involving fMRI of phonemic fluency with covert and overt responses (although undertaken with different task designs) (Phelps et al., 1997; Curtis et al., 1998; Dye et al., 1999; Hutchinson et al., 1999; Lurito et al., 2000; Fu et al., 2002; Abrahams et al., 2003; Halari et al., 2006). Showing reasonable consistency in the brain regions engaged during phonemic fluency, largely independent of the response mode, agrees with recent ERP findings (Perret and Laganaro, 2012) and provides important converging evidence that adds to scientific understanding of how word retrieval function and cognitive control function are distributed and work together in the brain at a gross level. However, as is typical of many preliminary fMRI studies, the cohort size of 12 individuals that was investigated in the present work limits the statistical power for detecting brain activity and behavior. The sample size ensured the negligible impact of certain experimental design choices that were made for expediency, such as requiring subjects to perform two runs of written phonemic fluency for the letter “S,” and inclusion of a small number of individuals who were not right-handed (but displayed left-lateralized brain activity) or non-native but highly fluent English speakers. For example, considering the latter factor, studies with much larger cohort sizes have revealed significant correlates in the medial brain structure with phonemic fluency (increased gray matter in the caudate nucleus) that is associated with suppressing the first language interacting with the second language used for the word retrieval task (Grogan et al., 2009). Such differences are much smaller than the individual subject variability in the present study. Regarding the response modality, it is expected in the future that differences in fMRI brain activity and behavior will be revealed for phonemic fluency with overt and written responses—but in keeping with the present study, such differences will be relatively small. To reveal these differences will be a demanding undertaking, given that multiple requirements must be satisfied together: (a) state-of-the-art fMRI data collection including real-time motion correction, correction for dynamic magnetic field inhomogeneity, as well as availability of fMRI-compatible tablet technology for recording written responses and state-of-the-art microphone technology for recording overt speech; (b) a large, homogeneous subject cohort; (c) extensive behavioral testing of written and overt phonemic fluency both inside and outside the MRI system, thus assessing the effect of fMRI on behavioral responses; and (d) sophisticated fMRI analysis, likely including single-subject optimized data pre-processing pipelines (Churchill et al., 2012) and data-driven multivariate methods to reveal subtle differences in the time-dependent patterns of brain activity associated with characteristic features of phonemic fluency, such as the early phase response, and word clustering strategies. Despite these collective challenges, we are optimistic that such a study can be undertaken in the near future.

## Conclusion

The present study demonstrates the applicability of fMRI-compatible tablet technology for studying brain activity related to language processing, using the example of a long-duration self-paced written version of phonemic fluency. Over 12 subjects, the brain activity for written phonemic fluency localized to regions similar to those found in fMRI studies using different methodology, involving covert and overt speech. Brain activity in the late phase vs. the early phase of written phonemic fluency was localized in the bilateral middle frontal and anterior cingulate gyri, associated with increased cognitive demands, such as initiation, maintenance, attention shifting and error processing, as task performance progressed in time. Given the difficulties to maintain fMRI data quality in tasks that require overt speech with free recall, written responses appear to provide a promising option for probing fluency networks. Other tablet-and-stylus-based fMRI approaches may be useful to study aspects of language production interacting with cognitive control in the frontal lobe.

### Conflict of interest statement

The authors declare that the research was conducted in the absence of any commercial or financial relationships that could be construed as a potential conflict of interest.
